# Supplementary material for: Urban Hedgehog Behavioural Responses to Temporary Habitat Disturbance versus Permanent Fragmentation
Source: Animals (Basel). 2020 Nov 13;10(11):2109. doi: 10.3390/ani10112109 (PMC7697271; doi:10.3390/ani10112109)
Supplement: Supplementary file 1 [file animals-10-02109-s001.zip › animals-982801/Table S1.docx]

Table S1: Overview of the study animals and data collected per animal.

| **animal_ID** | **sex** | **study side** | **startdate DD.MM.YYYY** | **enddate DD.MM.YYYY** | **number of investigated days** |
| --- | --- | --- | --- | --- | --- |
| 2016_01 | male | Treptower Park | 09.08.2016 | 19.09.2016 | 41 |
| 2016_02 | female | Treptower Park | 09.08.2016 | 19.09.2016 | 41 |
| 2016_08 | female | Treptower Park | 09.08.2016 | 19.09.2016 | 41 |
| 2016_09 | male | Treptower Park | 09.08.2016 | 12.09.2016 | 34 |
| 2016_13 | female | Treptower Park | 09.08.2016 | 19.09.2016 | 41 |
| 2016_17 | female | Treptower Park | 09.08.2016 | 19.09.2016 | 41 |
| 2016_19 | male | Treptower Park | 09.08.2016 | 19.09.2016 | 41 |
| 2016_21 | male | Treptower Park | 09.08.2016 | 19.09.2016 | 41 |
| 2017_08 | male | Tierpark | 15.08.2017 | 13.09.2017 | 29 |
| 2017_19 | male | Tierpark | 15.08.2017 | 21.08.2017 | 6 |
| 2017_20 | female | Tierpark | 14.08.2017 | 04.09.2017 | 21 |
| 2017_26 | female | Tierpark | 15.08.2017 | 13.09.2017 | 29 |
| 2017_28 | female | Tierpark | 14.08.2017 | 04.09.2017 | 21 |
| 2017_31 | male | Tierpark | 15.08.2017 | 05.09.2017 | 21 |
| 2017_32 | female | Tierpark | 14.08.2017 | 13.09.2017 | 30 |
| 2017_35 | male | Tierpark | 15.08.2017 | 18.08.2017 | 3 |
