# Supplementary material for: Urban Hedgehog Behavioural Responses to Temporary Habitat Disturbance versus Permanent Fragmentation
Source: Animals (Basel). 2020 Nov 13;10(11):2109. doi: 10.3390/ani10112109 (PMC7697271; doi:10.3390/ani10112109)
Supplement: Supplementary file 1 [file animals-10-02109-s001.zip › animals-982801/Table S2.docx]

Table S2: Overview of mean yearly weather data (source: https://www.wetteronline.de/).

|  | **Mean temperature [^o^C]** | **Rain**  **[ l/m^2^]** |
| --- | --- | --- |
| August (mean of 4 reference years) | 17.8 | 59 |
| 2016-August | 18.7 | 40 |
| 2017-August | 19.0 | 75 |
|  |  |  |
| September (mean of 4 reference years) | 14.1 | 46 |
| 2016-September | 18.1 | 20 |
| 2017-September | 14.0 | 35 |
|  |  |  |
| Summer (mean of 4 reference years) | 17.7 | 182 |
| 2016 summer | 19.4 | 180 |
| 2017 summer | 18.8 | 420* |
|  |  |  |
| total year (mean of 4 reference years) | 9.1 | 573 |
| 2016 year | 10.5 | 505 |
| 2017 year | 10.4 | 815 |

*high value is mainly caused by trough “Raimund” in June 2016
