# Supplementary material for: Urban Hedgehog Behavioural Responses to Temporary Habitat Disturbance versus Permanent Fragmentation
Source: Animals (Basel). 2020 Nov 13;10(11):2109. doi: 10.3390/ani10112109 (PMC7697271; doi:10.3390/ani10112109)
Supplement: Supplementary file 1 [file animals-10-02109-s001.zip › animals-982801/Table S3.docx]

Table S3: Overview about model results.

### MCP

Linear Model and Outcome

lme_mcp <- lme(mcp95_area ~ treatment*sex, random = ~ 1|animal_id, res.new,

weights = varPower())

Anova(lme_mcp)

## Analysis of Deviance Table (Type II tests)

## Response: mcp95_area

## Chisq Df Pr(>Chisq)

## treatment 54.8210 2 1.247e-12 ***

## sex 6.4797 1 0.01091 *

## treatment:sex 1.7432 2 0.41828

## ---

## Signif. codes: 0 '***' 0.001 '**' 0.01 '*' 0.05 '.' 0.1 ' ' 1

summary(glht(mod_mcp_plot, linfct = mcp(inter_Treat_Sex = K)))

## Simultaneous Tests for General Linear Hypotheses

## Multiple Comparisons of Means: User-defined Contrasts

## Fit: lme.formula(fixed = mcp95_area ~ inter_Treat_Sex, data = res.new,

## random = ~1 | animal_id, weights = varPower())

##

## Linear Hypotheses:

## Estimate Std. Error z value Pr(>|z|)

## pre-festival_m - pre-festival_f == 0 1.8968 0.6891 2.753 0.0327*

## festival_f - pre-festival_f == 0 -1.0290 0.2214 -4.648 <0.001***

## fragmented_f - pre-festival_f == 0 1.9421 0.6945 2.796 0.0291*

## festival_m - pre-festival_m == 0 -1.4665 0.3770 -3.890 <0.001***

## fragmented_m - pre-festival_m == 0 0.7302 0.8663 0.843 0.9200

## fragmented_m - fragmented_f == 0 0.6849 0.8706 0.787 0.9389

## ---

## Signif. codes: 0 '***' 0.001 '**' 0.01 '*' 0.05 '.' 0.1 ' ' 1

## (Adjusted p values reported -- single-step method)

## # A tibble: 6 x 8

## inter_Treat_Sex mean_mcp sd_mcp n_mcp lower upper sex treatment

## <fct> <dbl> <dbl> <int> <dbl> <dbl> <fct> <fct>

## 1 pre-festival_f 2.55 1.32 77 2.25 2.85 f pre-festival

## 2 festival_f 1.76 1.32 81 1.47 2.05 f festival

## 3 fragmented_f 4.73 2.96 78 4.06 5.39 f fragmented

## 4 pre-festival_m 4.71 2.79 79 4.09 5.34 m pre-festival

## 5 festival_m 3.08 2.34 71 2.53 3.63 m festival

## 6 fragmented_m 5.46 4.52 40 4.01 6.90 m fragmented

### KDE

mod_kde <- lme(kde50_area ~ treatment*sex, random = ~ 1|animal_id, res.new,

weights = varPower())

car::Anova(mod_kde)

## Analysis of Deviance Table (Type II tests)

## Response: kde50_area

## Chisq Df Pr(>Chisq)

## treatment 44.9871 2 1.703e-10 ***

## sex 10.2643 1 0.001356 **

## treatment:sex 5.6387 2 0.059644 .

Speed [m/s]

mod_speed <- lmer(sqrt(speed) ~ treatment*sex + (1 |animal_id), mydata.sf.EOBS)

Anova(mod_speed)

mod_speed_res <- multcomp::glht(mod_speed, linfct = multcomp::mcp(treatment = "Tukey"), interaction_average = FALSE, covariate_average = TRUE)

Simultaneous Tests for General Linear Hypotheses

## Multiple Comparisons of Means: Tukey Contrasts

## Fit: lmer(formula = sqrt(speed) ~ treatment * sex + (1 | animal_id),

## data = mydata.sf.EOBS)

## Linear Hypotheses:

## Estimate Std. Error z value Pr(>|z|)

## festival - pre-festival == 0 -0.005701 0.001715 -3.325 0.00206 **

## fragmented - pre-festival == 0 0.025613 0.008429 3.038 0.00511 **

## fragmented - festival == 0 0.031314 0.008425 3.717 < 0.001 ***

## ---

## Signif. codes: 0 '***' 0.001 '**' 0.01 '*' 0.05 '.' 0.1 ' ' 1

## (Adjusted p values reported -- single-step method)

# A tibble: 3 x 7

## treatment mean.speed sd.speed n.speed lower upper

## 1 pre-fest~ 0.0404 0.0365 9600 0.0396 0.0411

## 2 festival 0.0384 0.0353 9295 0.0376 0.0391

## 3 fragment~ 0.0485 0.0442 6493 0.0475 0.0496

## # ... with 1 more variable: geom <MULTIPOINT [m]>

Search intensity

res.new$distance_traveld/(res.new$mcp95_area*10000) [m/m²*d]

**Turning angles**

loop for pre-festival vs. festival

n = 1000

result <- data.frame(p_value = rep(NA,n), valueW = NA)

for (i in 1:n){

hmm.new <- hmm_data %>%

group_by(ID) %>%

sample_n(170)

result.test <- ks.test(abs(hmm.new$angle[hmm.new$treatment== "pre-festival"]) ,abs(hmm.new$angle[hmm.new$treatment== "festival"]))

result$p_value[i] <- result.test$p.value

result$valueW[i] <- result.test$statistic

}

result_pre_fest <- result

mean(result_pre_fest$p_value)

0.1138248

### Centroid shift

Normalize the data

res.fest$mean_x <- mean(res.fest$centroid_x[res.fest$treatment == "pre-festival"])

res.fest$mean_y <- mean(res.fest$centroid_y[res.fest$treatment == "pre-festival"])

res.fest$change_x <- abs(res.fest$mean_x - res.fest$centroid_x)

res.fest$change_y <- abs(res.fest$mean_y - res.fest$centroid_y)

mod_nom_x <- lmer(sqrt(change_x) ~ treatment*sex + (1 |animal_id), res.fest)

car::Anova(mod_nom_x)

## Analysis of Deviance Table (Type II Wald chisquare tests)

## Response: sqrt(change_x)

## Chisq Df Pr(>Chisq)

## treatment 80.5897 1 < 2.2e-16 ***

## sex 0.3572 1 0.55

## treatment:sex 21.4375 1 3.655e-06 ***

mod_nom_y <- lmer(sqrt(change_y) ~ treatment*sex + (1 |animal_id), res.fest)

car::Anova(mod_nom_y)

## Analysis of Deviance Table (Type II Wald chisquare tests)

## Response: sqrt(change_y)

## Chisq Df Pr(>Chisq)

## treatment 80.858 1 < 2.2e-16 ***

## sex 0.886 1 0.3465542

## treatment:sex 11.790 1 0.0005954 ***

## Signif. codes: 0 '***' 0.001 '**' 0.01 '*' 0.05 '.' 0.1 ' ' 1

car::Anova(mod_nom_x)

## Analysis of Deviance Table (Type II Wald chisquare tests)

## Response: sqrt(change_x)

## Chisq Df Pr(>Chisq)

## treatment 80.5897 1 < 2.2e-16 ***

## sex 0.3572 1 0.55

## treatment:sex 21.4375 1 3.655e-06 ***

## Signif. codes: 0 '***' 0.001 '**' 0.01 '*' 0.05 '.' 0.1 ' ' 1

car::Anova(mod_nom_y)

## Analysis of Deviance Table (Type II Wald chisquare tests)

## Response: sqrt(change_y)

## Chisq Df Pr(>Chisq)

## treatment 80.858 1 < 2.2e-16 ***

## sex 0.886 1 0.3465542

## treatment:sex 11.790 1 0.0005954 ***

## Signif. codes: 0 '***' 0.001 '**' 0.01 '*' 0.05 '.' 0.1 ' ' 1

### Behaviour

#### Balled up

car::Anova(glmer(cbind(bu, all - bu) ~ treatment*sex + (1 | hedgehog.id),

data = daily.data,

family = binomial))

Analysis of Deviance Table (Type II Wald chisquare tests)

## Response: cbind(bu, all - bu)

## Chisq Df Pr(>Chisq)

## treatment 598.4354 2 < 2.2e-16 ***

## sex 0.4289 1 0.512551

## treatment:sex 11.8406 2 0.002684 **

## ---

## Signif. codes: 0 '***' 0.001 '**' 0.01 '*' 0.05 '.' 0.1 ' ' 1

summary(glht(gm1, linfct = mcp(inter_Treat_Sex = K)))

## Simultaneous Tests for General Linear Hypotheses

## Multiple Comparisons of Means: User-defined Contrasts

## Fit: glmer(formula = cbind(bu, all - bu) ~ inter_Treat_Sex + (1 |

## hedgehog.id), data = daily.data, family = binomial)

## Linear Hypotheses:

## Estimate Std. Error z value Pr(>|z|)

## pre-festival_m - pre-festival_f == 0 0.03941 0.25135 0.157 1.000

## festival_f - pre-festival_f == 0 0.15268 0.01015 15.040 <1e-04***

## festival_m - pre-festival_m == 0 0.20006 0.01022 19.571 <1e-04 ***

## fragmented_f - pre-festival_f == 0 0.20941 0.25133 0.833 0.929

## fragmented_m - pre-festival_m == 0 -0.12687 0.25162 -0.504 0.992

## fragmented_m - fragmented_f == 0 -0.29687 0.25160 -1.180 0.755

##

## Signif. codes: 0 '***' 0.001 '**' 0.01 '*' 0.05 '.' 0.1 ' ' 1

## (Adjusted p values reported -- single-step method)

#### Walking

car::Anova(glmer(cbind(w, all - w) ~ treatment*sex + (1 | hedgehog.id),

data = daily.data,

family = binomial))

## Analysis of Deviance Table (Type II Wald chisquare tests)

## Response: cbind(w, all - w)

## Chisq Df Pr(>Chisq)

## treatment 9.5608 2 0.008393 **

## sex 0.0205 1 0.886235

## treatment:sex 1.9247 2 0.381989

## ---

## Signif. codes: 0 '***' 0.001 '**' 0.01 '*' 0.05 '.' 0.1 ' ' 1

## Simultaneous Tests for General Linear Hypotheses

## Multiple Comparisons of Means: User-defined Contrasts

## Fit: glmer(formula = cbind(w, all - w) ~ inter_Treat_Sex + (1 | hedgehog.id),

## data = daily.data, family = binomial)

## Linear Hypotheses:

## Estimate Std. Error z value Pr(>|z|)

## pre-festival_m - pre-festival_f == 0 0.10452 0.17186 0.608 0.9812

## festival_f - pre-festival_f == 0 0.01557 0.01133 1.374 0.6211

## festival_m - pre-festival_m == 0 0.02970 0.01110 2.675 0.0419 *

## fragmented_f - pre-festival_f == 0 0.27632 0.17184 1.608 0.4536

## fragmented_m - pre-festival_m == 0 0.02479 0.17218 0.144 1.0000

## fragmented_m - fragmented_f == 0 -0.14701 0.17215 -0.854 0.9220

##

## Signif. codes: 0 '***' 0.001 '**' 0.01 '*' 0.05 '.' 0.1 ' ' 1

## (Adjusted p values reported -- single-step method)

#### Resting

## Analysis of Deviance Table (Type II Wald chisquare tests)

## Response: cbind(ar, all - ar)

## Chisq Df Pr(>Chisq)

## treatment 203.2121 2 < 2.2e-16 ***

## sex 0.2557 1 0.6131

## treatment:sex 54.6765 2 1.34e-12 ***

## ---

## Signif. codes: 0 '***' 0.001 '**' 0.01 '*' 0.05 '.' 0.1 ' ' 1

##

## Simultaneous Tests for General Linear Hypotheses

## Multiple Comparisons of Means: User-defined Contrasts

## Fit: glmer(formula = cbind(ar, all - ar) ~ inter_Treat_Sex + (1 |

## hedgehog.id), data = daily.data, family = binomial)

## Linear Hypotheses:

## Estimate Std. Error z value Pr(>|z|)

## pre-festival_m - pre-festival_f == 0 -0.22498 0.22531 -0.999 0.860

## festival_f - pre-festival_f == 0 -0.20539 0.01335 -15.391 <0.001 ***

## festival_m - pre-festival_m == 0 -0.06227 0.01458 -4.269 <0.001 ***

## fragmented_f - pre-festival_f == 0 -0.45706 0.22532 -2.028 0.211

## fragmented_m - pre-festival_m == 0 0.08880 0.22570 0.393 0.997

## fragmented_m - fragmented_f == 0 0.32088 0.22571 1.422 0.587

## Signif. codes: 0 '***' 0.001 '**' 0.01 '*' 0.05 '.' 0.1 ' ' 1

## (Adjusted p values reported -- single-step method)

#### Other

# Analysis of Deviance Table (Type II Wald chisquare tests)

## Response: cbind(other, all - other)

## Chisq Df Pr(>Chisq)

## treatment 248.2961 2 <2e-16 ***

## sex 0.1481 1 0.7004

## treatment:sex 105.6829 2 <2e-16 ***

## ---

## Signif. codes: 0 '***' 0.001 '**' 0.01 '*' 0.05 '.' 0.1 ' ' 1

## Simultaneous Tests for General Linear Hypotheses

## Multiple Comparisons of Means: User-defined Contrasts

## Fit: glmer(formula = cbind(other, all - other) ~ inter_Treat_Sex +

## (1 | hedgehog.id), data = daily.data, family = binomial)

## Linear Hypotheses:

## Estimate Std. Error z value Pr(>|z|)

## pre-festival_m - pre-festival_f == 0 -0.048830 0.190356 -0.257 0.999679

## festival_f - pre-festival_f == 0 -0.040495 0.009461 -4.280 0.000107 ***

## festival_m - pre-festival_m == 0 -0.179777 0.009839 -18.272 < 1e-04 ***

## fragmented_f - pre-festival_f == 0 -0.260492 0.190355 -1.368 0.625203

## fragmented_m - pre-festival_m == 0 0.008588 0.190594 0.045 1.000000

## fragmented_m - fragmented_f == 0 0.220250 0.190591 1.156 0.770731

## ---

## Signif. codes: 0 '***' 0.001 '**' 0.01 '*' 0.05 '.' 0.1 ' ' 1

## (Adjusted p values reported -- single-step method)

### DFC

Simultaneous Tests for General Linear Hypotheses

Multiple Comparisons of Means: User-defined Contrasts

Fit: lmer(formula = value.data.asin ~ inter_Treat_Sex + (1 | animal_id),

data = df.data.new)

Linear Hypotheses:

Estimate Std. Error z value Pr(>|z|)

pre-festival_m - pre-festival_f == 0 0.05671 0.07889 0.719 0.95930

festival_f - pre-festival_f == 0 -0.10386 0.03569 -2.910 0.02053 *

festival_m - pre-festival_m == 0 -0.13514 0.03766 -3.589 0.00185 **

fragmented_f - pre-festival_f == 0 0.16917 0.08207 2.061 0.19341

fragmented_m - pre-festival_m == 0 -0.06732 0.09682 -0.695 0.96460

fragmented_m - fragmented_f == 0 -0.17978 0.09942 -1.808 0.31987

---

Signif. codes: 0 ‘***’ 0.001 ‘**’ 0.01 ‘*’ 0.05 ‘.’ 0.1 ‘ ’ 1

(Adjusted p values reported -- single-step method)
