# Supplementary figures and images for: Urban Hedgehog Behavioural Responses to Temporary Habitat Disturbance versus Permanent Fragmentation
Source: Animals (Basel). 2020 Nov 13;10(11):2109. doi: 10.3390/ani10112109 (PMC7697271; doi:10.3390/ani10112109)

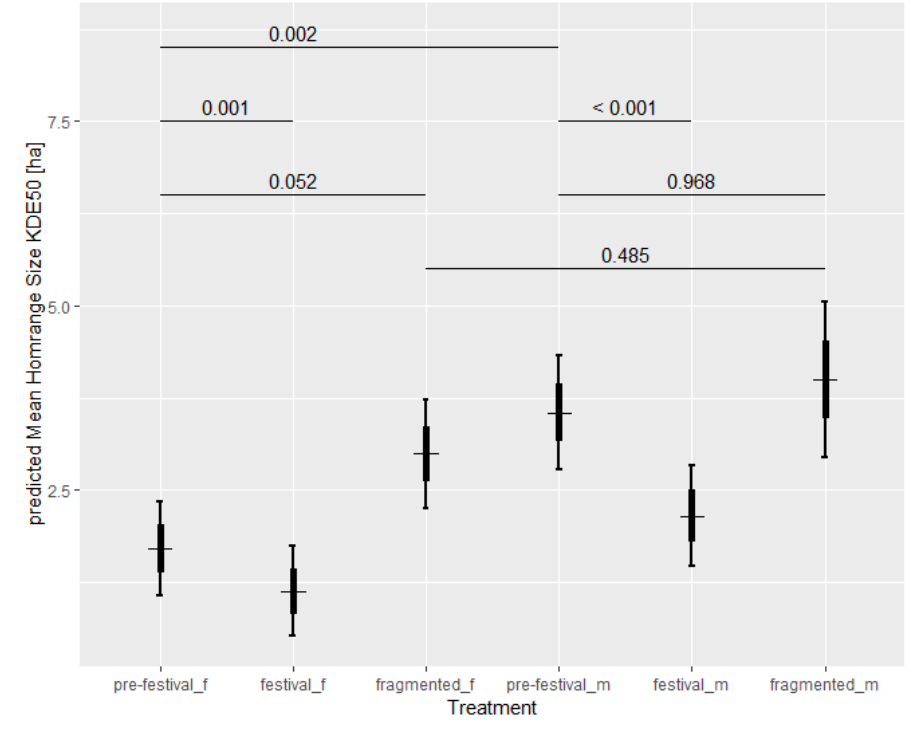

Supplement: Supplementary file 1 [file animals-10-02109-s001.zip › animals-982801/Figure S1.png]

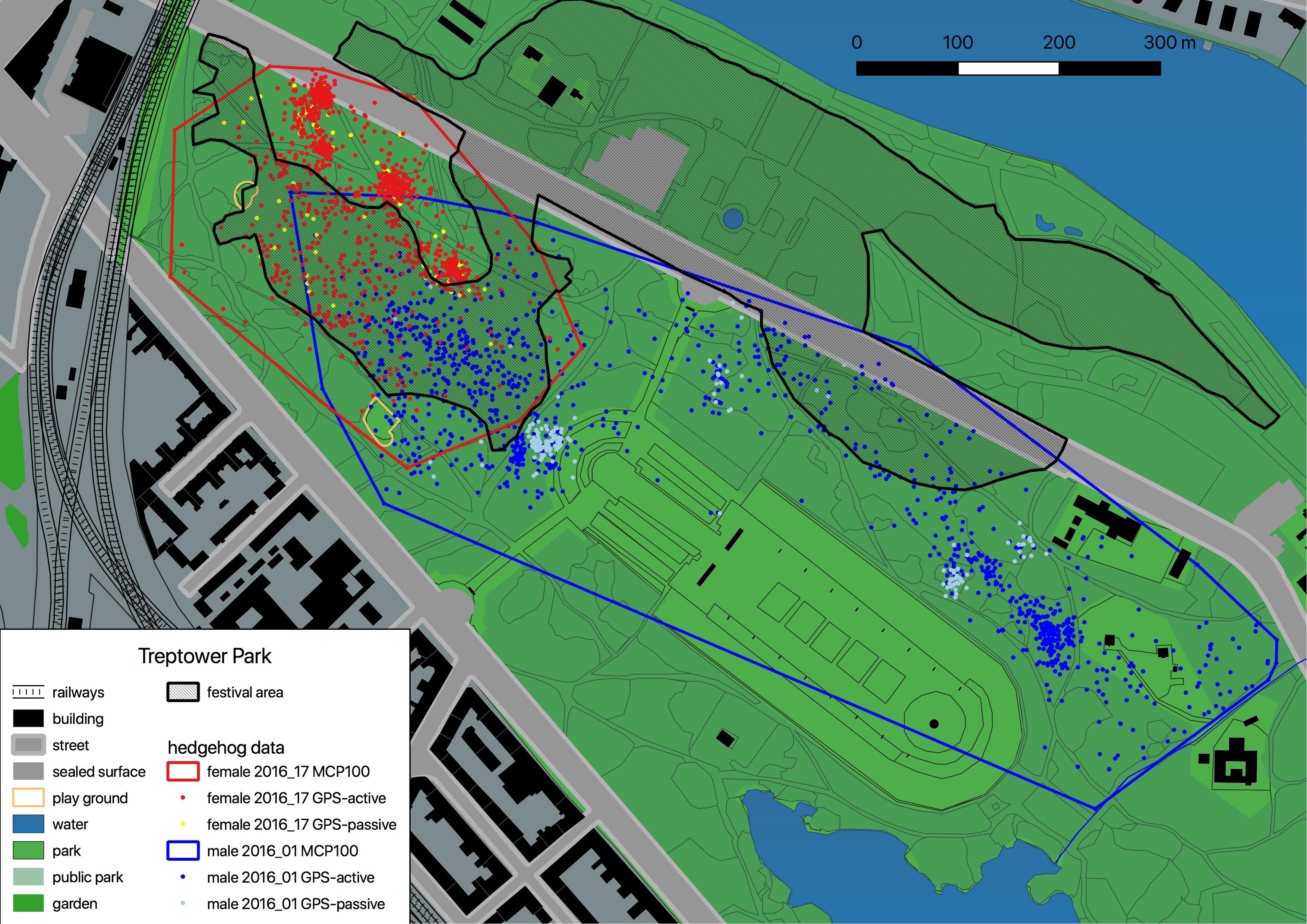

Supplement: Supplementary file 1 [file animals-10-02109-s001.zip › animals-982801/Figure S2.jpg]

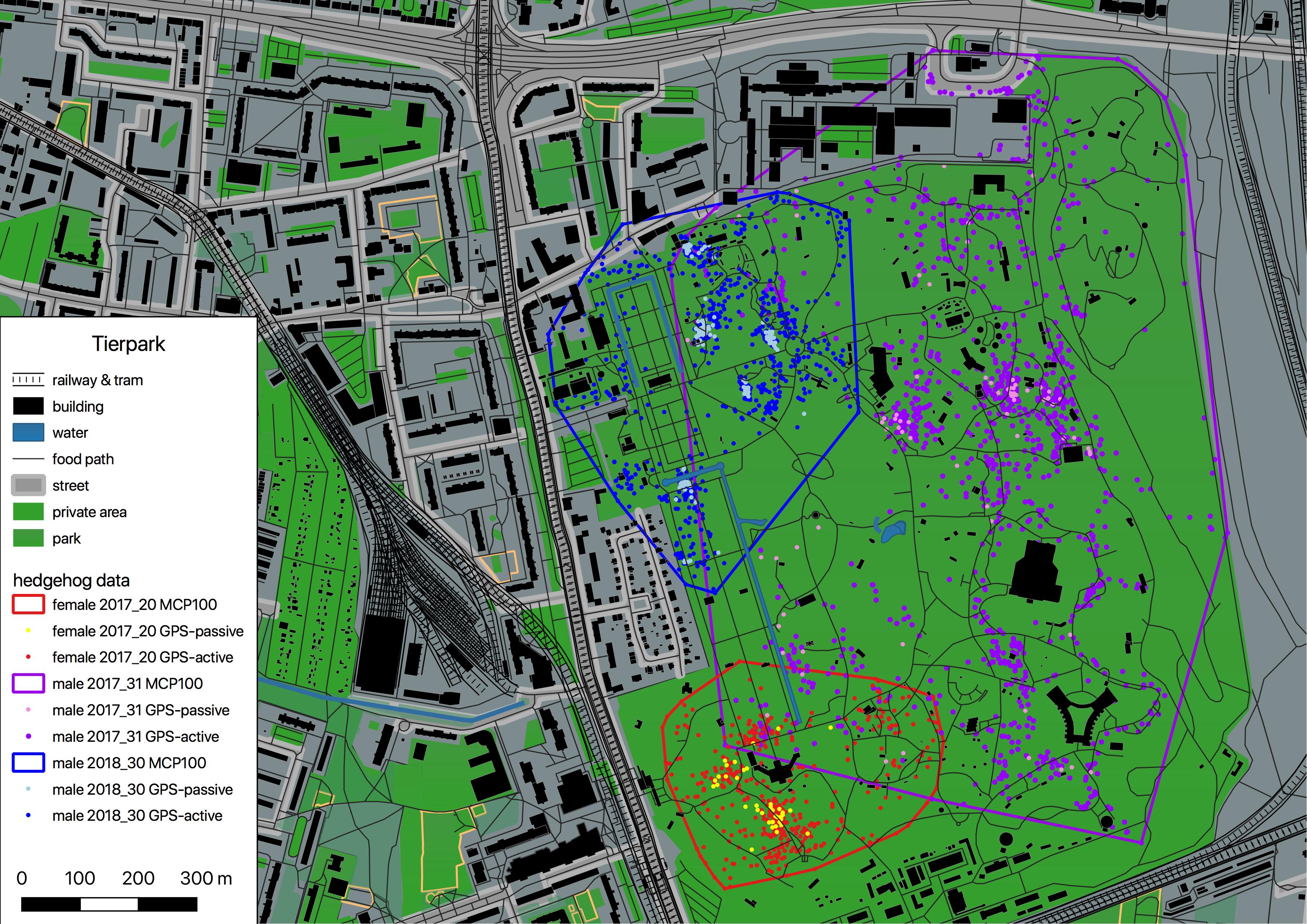

Supplement: Supplementary file 1 [file animals-10-02109-s001.zip › animals-982801/Figure S3.jpg]
